# Electrochemical Peptide-Based Sensors for Foodborne Pathogens Detection

**DOI:** 10.3390/molecules26113200

**Published:** 2021-05-27

**Authors:** Mihaela Tertis, Oana Hosu, Bogdan Feier, Andreea Cernat, Anca Florea, Cecilia Cristea

**Affiliations:** Department of Analytical Chemistry, Iuliu Hațieganu University of Medicine and Pharmacy, 4 Louis Pasteur Street, 400349 Cluj-Napoca, Romania; mihaela.tertis@umfcluj.ro (M.T.); Hosu.Oana@umfcluj.ro (O.H.); Feier.George@umfcluj.ro (B.F.); Ilioaia.Andreea@umfcluj.ro (A.C.); Anca.Florea@umfcluj.ro (A.F.)

**Keywords:** peptide, electrochemical sensors, food safety, food contamination, quality control

## Abstract

Food safety and quality control pose serious issues to food industry and public health domains, in general, with direct effects on consumers. Any physical, chemical, or biological unexpected or unidentified food constituent may exhibit harmful effects on people and animals from mild to severe reactions. According to the World Health Organization (WHO), unsafe foodstuffs are especially dangerous for infants, young children, elderly, and chronic patients. It is imperative to continuously develop new technologies to detect foodborne pathogens and contaminants in order to aid the strengthening of healthcare and economic systems. In recent years, peptide-based sensors gained much attention in the field of food research as an alternative to immuno-, apta-, or DNA-based sensors. This review presents an overview of the electrochemical biosensors using peptides as molecular bio-recognition elements published mainly in the last decade, highlighting their possible application for rapid, non-destructive, and in situ analysis of food samples. Comparison with peptide-based optical and piezoelectrical sensors in terms of analytical performance is presented. Methods of foodstuffs pretreatment are also discussed.

## 1. Introduction

Foodborne pathogens cause over 600 million maladies worldwide, mainly being associated with the living conditions and, therefore, having a higher prevalence in countries with a low/middle income. The globalization process determined the development of international/intercontinental food supply chains that are directly exposed to contamination in various stages, from primary sources to restaurants and shopping centers, especially when the market demands require storage for long periods of time [1].

The diseases caused by bacteria are difficult to be detected in early stages and could be fatal in some cases, especially in children, elders, or patients undergoing immunosuppressive treatments. This issue has become a constantly growing health problem and causes about 30% of the foodborne-caused deaths among children under the age of 5 [2]. Thus, food safety and security have become major global concerns [3].

The most commonly microorganisms involved in foodborne-caused illnesses are represented by bacteria (Salmonella, Listeria, *Vibrio cholerae*, *Escherichia coli*) and viruses (Norovirus and Hepatitis A virus); the contamination vehicles are mainly animal products, vegetables, and water [4].

The conventional methods used for their detection depend on the nature of the contaminant and are represented by culture plating, polymerase chain reaction (PCR), and enzyme-linked immunosorbent assays (ELISA). Although these methods are well known for their high specificity, the results can take from several hours to 1–2 days, and they involve complicated procedures and high costs. Thus, the need of monitorization of pathogen content with rapid, specific, efficient, and portable devices enables the development of electrochemical multiplexed sensing systems [5].

The electrochemical sensors are designed by coupling a receptor to an electrochemical transducer that translates the analytical information generated by the analyte–electrode electrochemical interaction into a measurable electrical signal. The majority of the electrochemical sensors are based on modified electrodes with different types of molecules that ensure the specificity toward a designated target. Enzymes [6], cells [7], antibodies [8], aptamers [9,10], antigens [11], and peptides [12] are some of the bio-compounds that can be immobilized onto the electrodes’ surface in order to detect a single compound from complex matrices.

Particularly, the elaboration of platforms for the design of biomedical devices requires multidisciplinary know-how regarding the integration of the biomolecule into an inorganic platform without having secondary effects on the biological activity and achieving the maximum analytical performances. The literature from the last decade describes the use of proteins and peptides as state-of-the-art approaches for biosensors with exhaustive applications. Peptides and proteins have a structure formed by amino acids (AA) that are linked via peptide bonds, the main difference between them being the greater number of AA for proteins (over 50 AA) as compared to peptides. In addition, peptides are characterized by a high affinity for organic/inorganic compounds, a well-known structure, chemical stability, biocompatibility, low immunogenicity, and do not imply complicated protocols and high synthesis costs, at least for the ones having maximum 10 AA. These are good premises for their successful integration in the development of sensing devices for medical purposes [13,14,15,16].

Peptides can be classified as natural, the ones that can be found in the human body such as hormones, neurotransmitters, or immunomodulators, and artificial/synthetic, which are de novo synthesized compounds via computational methods or in vitro assessment.

The literature studies indicated that peptides, regardless of their synthesis, have the capacity to recognize specific targets and were thoroughly used in the development of vaccines, diagnostic agents, and therapy agents for infectious diseases such as influenza, also known as the traditional flu, chronic hepatitis B, acquired immunodeficiency syndrome (AIDS), severe acute respiratory syndrome (SARS), and lately the coronavirus disease (COVID-19) [17]. The peptides used in therapy can block the viral attachment, the gene release especially in respiratory disease, and the viral protein assembly in SARS and COVID-19. The development of peptide-based vaccines aims to achieve the activation of B and/or T cells to promote an immune response [18].

Giving the actual pandemic context and the worldwide healthcare collapse regardless of the economical rating of the countries, the necessity to develop safe and efficient therapies or vaccines became the main concern of the biomedical world in order to stop the uncontrollable spreading of SARS-CoV-2 virus. Even though the outbreak started at the end of 2019, the structure of the virus was extensively studied based on previous research on SARS-CoV-1 virus, which was responsible for the SARS epidemic that affected more than 8000 patients and had 10% mortality. It was proved that the second version has 79% genetic similarities but uses the host’s angiotensin-converting enzyme 2 (ACE2) receptor for cellular penetration, and the new structure of protein S 2019’s version increases the pathogeny of the virus [18]. None of the actual therapies are peptide-based, but based on SARS-CoV-1 studies, several molecules derived from protein S that target the ACE2 receptor have perspectives to be implemented [19]. Regarding the diagnosis part, in addition to the PCR technique, a peptide from the S protein showed high specificity to SARS-CoV-2, which is a feature that can be exploited via electrochemical methods, and specifically the development of affinity-based sensors. Thus, in one example, serum samples were treated with the biotinylated S peptide conjugated with streptavidin magnetic beads and the detection of immunoglobulin G (IgG) and immunoglobulin M (IgM) SARS-CoV-2 antibodies was achieved by luminescence with an accuracy of 71.4% and 57.2% [20].

Herein, we present an extensive study regarding the use of peptides as recognition elements for the development of electrochemical sensing devices that have the ability to bring an innovative aspect toward the conventional detection approaches for various molecules involved in food safety and food quality control. A comparison with peptide-based optical and piezoelectrical sensors in terms of analytical performance toward foodborne pathogens is presented to highlight the advantages and disadvantages of the electrochemical peptide-based sensors.

The use of peptides in the development of sensors for food quality control has seen an important development, especially in recent years. This aspect can also be highlighted by the large number of publications (scientific articles and review studies) that have been published recently on this topic. A simple search in the database, using the keywords: “peptide-based sensors for food” obtained 20,445 results, out of which approximately 25% refer to electrochemical sensors. Thus, the topic addressed in this review can be justified as being important.

Although there are other review-type articles published to date that deal with the applications of peptides in sensors and biosensors, to the best of our knowledge, this study is the first overview that focuses only on the electrochemical biosensors using peptides as molecular bio-recognition elements applied for food samples analyses. The most important review articles were mentioned throughout our study; some present an overview of the various types of (bio)sensors based on peptides for their analytical use, along with significant advances over the last several years in related technologies [21] or describe the peptide-based biosensors and the role of peptides in the sensing process [22].

## 2. Peptides

### 2.1. Definition

**Figure 1:**
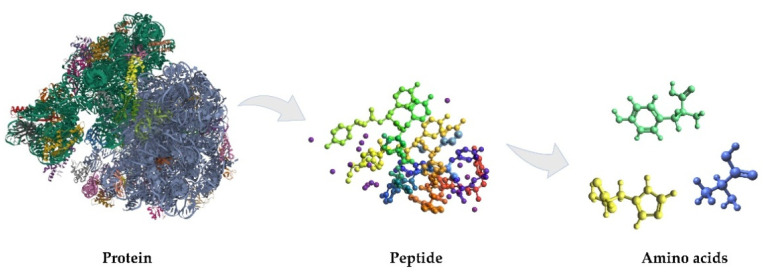
Structural composition of proteins and peptides.

Peptides are biomolecules with important roles in the organism. Multiple peptide chains form a protein unit (Figure 1). Peptides are short chains of 2 to 50 amino acids linked by peptide bonds, which are amides derived from two or more amino carboxylic acid molecules (the same or different) by the formation of a covalent bond from the carbonyl carbon of one AA to the nitrogen atom of another with formal loss of water [23]. Usually, the term refers to structures obtained using as building blocks the twenty natural α-amino acids, but it includes those derived from any amino carboxylic acid. All peptides except cyclic peptides have a *N*-terminal (amine group) and *C*-terminal (carboxyl group) residue at each end of the peptide. The amino acids have the same basic construction and vary only in the R-group at the central carbon (C) position of the molecule, which influences the configurations the peptides adopt, based on which R-groups are closely positioned in a specific peptide chain [21].

### 2.2. Classification of Peptides

There are many kinds of peptides, differing in many aspects. They can be classified according to their size, structure, properties, origin, functions, or applications.

Peptides containing fewer than ten or fifteen AA are called oligopeptides, and they include dipeptides, tripeptides and tetrapeptides.

Based on the amino acids present in the sequence and their secondary structure, the peptides can adopt an α-helix structure, a β-sheet, or other regular structures, including helical structures or turn structures, in which the backbone changes direction. The stability of the secondary structure of peptides is assured by the hydrogen bonds, but other interactions (disulfide bonds, cross-links, salt bridges, hydrophobic interactions, π stacking) can influence the stability of the peptides [24].

Based on the physical and chemical properties, peptides can be classified as cationic, amphipathic, and hydrophobic. Cationic peptides have a positive charge at physiological pH and are mostly composed of arginine, lysine, and histidine. Amphipathic peptides have hydrophilic and lipophilic regions that aid transport through biological membranes. They are classified in primary amphipathic, secondary amphipathic α-helical, β-sheet amphipathic, and proline-rich amphipathic peptides. Hydrophobic peptides have low net charge and are important for internalization into the membrane core [25].

According to their origin, peptides can be classified in natural and artificial peptides [26,27]. The natural peptides (e.g., hormones, neurotransmitters, growth factors), similar to the proteins, are produced via the messenger ribonucleic acid (mRNA), each amino acid being coded by a triplet of nucleosides in the ribosome, which is a large complex of RNA and proteins in the cell. There is synthesis outside the ribosome (non-ribosomal synthesis) of peptides too, involving enzymes capable of coupling to specific residues [28]. Natural peptides are suitable as starting points for pharmacophores or the design of drug-like molecules with incorporated secondary structural elements [24].

The artificial peptides present several desirable properties for the development of selective biosensors, such as high affinity to particular analytes (proteins included), high stability, easy modification, and large structure versatility, the specific sequences being obtained by matured synthesis protocols, by the screening and optimization of artificial peptides libraries [22].

Peptide-based nanomaterials (ranging from 20 to 200 nm) also promote the passive tumor targeting due to the enhanced permeability and retention effect in tumor tissues. Moreover, they can selectively adhere to targeted tumor cells by recognition of overexpressed enzymes in the tumoral microenvironment and can participate in the regulation of tumor cells’ apoptosis. Thus, the development of cell-penetrating peptides that have the ability to accumulate in tumors or intervene via other mechanisms is a major point in the diagnosis/treatment of malignant conditions [14].

A different class is represented by antimicrobial peptides that are recognition elements for bacteria and have the potential to be used as innovative therapeutic tools. These peptides have 6–50 AA, an amphiphilic nature, and demonstrate affinity for negatively charged bacteria. The antibacterial mechanisms are unclear, but one of the theory states that the cell membrane is altered by the disruption of the cytoplasmic membrane affecting the viability of the microorganism [29].

### 2.3. Advantages of Using Peptides in Sensors

Peptides are shorter than proteins and have the advantage that the peptide chain can specifically interact with cells, proteins, and enzymes improving the selectivity of the sensor toward designated targets. The 20 natural AAs determine the final structure of the peptides, and by engineering their order, one can achieve a specific configuration. The fact that they are electrostatically charged and their conductive properties enable the premises to be included in the design of electrochemical sensors and also to be associated with nanomaterials [30].

Peptides are versatile molecules that can self-assemble in different nanostructures controlled by non-covalent bonds such as electrostatic interactions, hydrogen bonds, and van der Waals interactions [15]. One such example is represented by the self-assembly of peptides onto graphene via π-interactions without affecting the physical and chemical properties of the surface but enhancing the electron transfer rate between the analyte and the electrodes’ surface [16]. This property is directly related to the length of the peptide chain: short peptides are flexible, can interconvert between conformations and form loosely packed films, while longer ones with a helicoidal configuration form well-ordered and densely packed films [15].

Peptides can be easily prepared with arbitrary sequences using standard solid phase peptide synthesis protocols, with fluorenylmethyloxycarbonyl (Fmoc) or tert-butyloxycarbonyl (t-Boc) as amine-protecting groups in order to prevent undesirable side reactions with various amino acid side chains. The solid phase consists of polymeric resin functionalized with reactive groups that link to the nascent peptide chain, allowing the peptide to be covalently attached to the support, while the reagents and other reaction products can be removed by successive washing and filtration steps. The same protocol allows for a wide range of functional molecules to be attached at the two terminal positions of a peptide sequence, while the peptides modified in a specific manner retain their high affinity to the target analyte [22,31].

An important advantage of peptides is that their particular structure and properties allow their use in many applications, such as of cyto-, enzymatic, apta-, geno-, and immuno-sensors. The binding of the analyte by the peptides generally does not directly generate a measurable signal, and therefore, conjugation with a signal marker (label) is an efficient strategy to quantify the bound analyte. The peptide-based biosensors usually involve the modification of the specific peptide sequence with a label in accordance with the analytical method used, a spacer that confers flexibility to the peptide and promotes accessibility to the analyte, and a chemical moiety (i.e., thiol, amino groups) that assures the peptide immobilization at the surface of the sensor [21].

Another advantage of peptides is that they present many physiological or therapeutic functions, from peptide hormones to anticancer treatments. The peptides can interact with the membrane by disrupting it, by passing through it, or by residing at the membrane interface.

There are also some membrane-active peptides that can fight against bacteria, fungi, parasites, and viruses by disrupting their membrane integrity or by inhibiting some functions of the cell. They consist of less than 100 amino acids and are promising candidates for the development of new drug leads. The cell-penetrating peptides (protein-transduction domain) are diverse membrane-active peptides that have less than 30 residues, which are usually negatively charged. They facilitate the delivery of different biomolecules across the cellular membrane, such as plasmid DNA, oligonucleotides, short interfering RNA, peptide nucleic acids, proteins, imaging agents, drugs, and liposomes [25].

Since the peptides have the same building block as proteins, there are situations in which a specific sequence can substitute a protein in biological analysis. It was demonstrated that synthetic peptides with specific sequences that can be obtained by screening and optimization of artificial peptide libraries can exhibit high affinity to targets [22]. Other advantages such as stability, easy synthesis and modification protocols, as well as chemical versatility successfully recommend peptides for applications in the field of sensors and biosensors.

When compared to antibodies and aptamers, peptides are advantageous because they are derived from natural sources with wide availability, can be easily selected from the available databases according to the target analyte envisaged for detection, and once the right sequence is chosen, it can be easily synthesized at a low cost. Furthermore, the fabrication, testing, and storage of sensors is favorable in the case of low molecular weight peptides that are more stable and flexible than antibodies [29,32]. This particular type of peptides retains the affinity toward bacteria at elevated temperatures or even when chemical denaturants occur [33,34], which significantly affect in a positive manner the stability and reproducibility of sensors. Furthermore, the high affinity of peptides for bacteria allows sensor operation at low bacteria concentrations [29,35].

## 3. Peptides in Sensors Design

According to the International Union of Pure and Applied Chemistry (IUPAC), a chemical sensor is defined as “a device that transforms chemical information, ranging from the concentration of a specific sample component to total composition analysis, into an analytically useful signal” [36], with the chemical information originating from a chemical reaction involving the analyte or from a physical property of the system investigated. The chemical sensors are made by connecting two functional units: the receptor and the transducer. At the receptor level, the chemical information is transformed into a suitable form of energy, and the transducer is capable of transforming the energy carrying the chemical information about the sample into a useful analytical signal. The transducer presents no selectivity, the modification of the receptor assuring the desired selectivity of the analysis [36].

According to the type of the employed transducer, the chemical sensors can be divided into electrical, magnetic, thermometric, mass sensitive, optical, and electrochemical sensors. The latter two types are the most used types of sensors. The sensors can also be classified according to the receptor component in physical, chemical, and biochemical sensors [36].

The electrochemical sensors are based on the transformation of the electrically stimulated or spontaneous (at zero-current conditions) electrochemical interaction of the analyte with the surface of the electrode into a useful signal. Several subgroups of the electrochemical sensors can be distinguished as voltamperometric sensors, potentiometric sensors, chemically sensitized field effect transistors, and potentiometric solid electrolyte gas sensors [36,37].

The electrochemical biosensors are based on similar principles as the chemical sensors, and they have been developed in order to improve the selectivity and sensitivity of the analyses. They could be defined as analytical devices that associate a highly selective or even specific biological recognition element with a suitable transduction method so that a useful analytical signal is obtained when the interaction between that bio-element and a target species occurs [38]. Many bio-elements can be used for the development of the electrochemical biosensors, from enzymes to whole cells, each type of bio-element requiring an adaptation of the electrode modification and analysis methods. A promising direction in the development of biosensors is represented by biomimetic sensors, which are adaptable to a variety of target molecules, using molecularly imprinted polymers (MIPs). MIPs are artificial receptors obtained by the polymerization of a monomer in the presence of the analyte (acting as the template); after the polymerization step, the template is removed, leaving empty cavities that will be complementary in shape, size, and chemical functionalities to the analyte [39].

**Figure 2:**
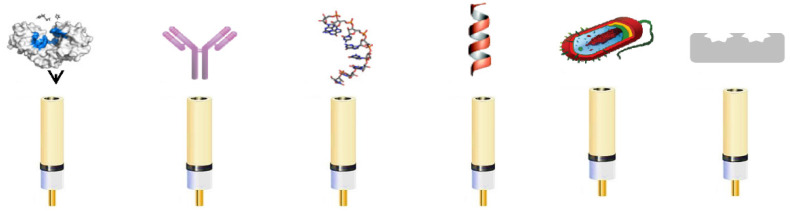
The biological recognition elements used for electrochemical biosensors development (from left to right): enzymes, antibodies, aptamers, peptides, whole cells, and MIP.

Figure 2 presents the different types of bio-elements used for the fabrication of biosensors: enzymes, antibodies, aptamers (short single stranded-DNA or RNA oligonucleotides, artificially selected for their capacity to bind specifically a target molecule), peptides, whole cells, and MIPs.

The electrochemical biosensors can be labeled with a redox probe, its electrochemical signal being measured before and after the biological element–analyte interaction or they can be label-free when the target is redox active or a suitable electrochemical technique (e.g., electrochemical impedance spectroscopy—EIS) can distinguish the signal modification due to the bioelement–analyte interaction.

There is a plethora of sensitive electrochemical techniques (potentiometry, chronoamperometry, voltammetry—differential pulse voltammetry and square-wave voltammetry—and EIS) that can be used with biosensors, allowing very low detection limits, in the range of micromolar to femtomolar.

The special properties of the peptides have led to the development of many peptide-based sensors in the recent decades for a wide range of applications. Thus, peptide-based sensors were developed for many applications in the biomedical field, for food safety monitoring, for food contamination problems with microbes and pathogens, as well as for the environment monitoring.

**Figure 3:**
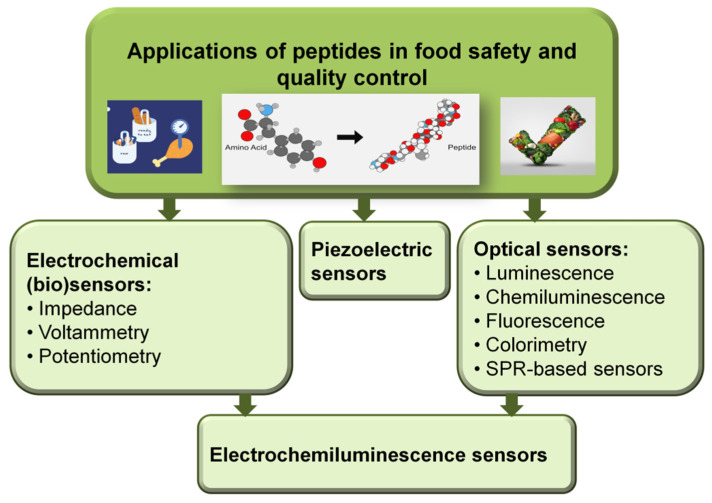
Overview of peptide-based sensors applications in food safety and quality control.

Thus, several analytical methods and detection strategies such as mass perturbance, optical and electrochemical ones were reported involving the use of peptides as sensing elements. A synthetic presentation of the analytical strategies in which the peptides are involved and which aim to ensure food quality and control is presented in Figure 3.

Due to the fact that the synthesis protocols for peptides are well known and mature and the peptides present high selectivity substrates for enzymes, these compounds can be considered real building blocks for the design of innovative biosensors in biological analysis. In this case, the signal to be measured is related to the bio-conjugation process that can provide an efficient way to convert the interaction information between peptides and analytes.

Furthermore, several fluorophores that can be linked to changes that can occur in the environment (the so-called environmentally-sensitive fluorophores) were used as labels for peptides for the elaboration of some very effective peptide-based molecular sensors. Other types of markers or labels that have often been reported as peptide-conjugated signal markers are noble metal nanoparticles, fluorescent polymers, and graphene-based materials or dyes.

Peptides have been intensively applied as bio-recognition elements for various analytes such as proteins, enzymes, nucleic acids, bacteria, or metal ions for the fabrication of highly selective and sensitive biosensors for relevant markers in the biomedical, food, and environmental fields [40,41].

Another important aspect that led to the development of the field of peptide-based sensors was related to the multiple possibilities of their immobilization process on the surfaces used in detection, whether it is electrodes, chips, piezoelements, or other surfaces involved in the detection process [21,22,42].

While the use of optical or piezoelectrical sensors is generally conditioned by a laboratory setting due to the complex equipment and other experimental requirements, electrochemical sensors based on peptides have experienced a high level of miniaturization and decentralization in the recent years, in this case highlighting the manufacturing of portable mini-sensors or biochips in the future. Miniaturized, portable, low-cost, fast and easy to use peptide-based chip-biosensors will certainly be the next step in the development of biomedical analysis and clinical diagnosis [43,44,45].

There are three different functions that peptides can possess/fulfill when involved in peptide-based sensors, namely the bio-recognition element (receptor), the linker–enzymatic substrate, and the framework. These functions will be further revealed using selected examples from different fields.

## 4. Food Samples Treatment Methods

Foodstuffs treatment before analysis is often employed in order to ensure homogenous analytes distribution and to reduce the analysis time, solvent use, and overall analysis costs.

Several challenges may be encountered when analyzing food samples:

- A specific physical state may be required for analysis;
- The constituents often generate an interfering matrix effect;
- The levels of analytes are very low;
- A decrease in sensitivity, low peak-to-peak separation, or changes in the peak shape compared to standard solutions analysis may be encountered.

Depending on the type of the food sample, a prior step such as washing or removing the surface or inner hard matter is required (e.g., soil, sand, skin or pit fruits, eggshell, bone, etc.). In order to avoid heterogeneous foodstuffs analysis, especially in semi-solid and solid samples, different methods are employed, such as (i) mechanical methods by grinding, mixing, slicing, or blending processes; (ii) enzymatic methods under the activity of proteases, cellulases, or lipases; (iii) chemical methods by the action of strong pH change (acid or alkaline) or by surfactants addition [46]. However, liquid samples may be subjected to dilution or concentration by evaporation or even no prior treatment.

After homogenization, smaller aliquots of samples are further subjected for analysis. Hence, compared to traditional analytical techniques, little consumption of solvents and low volume of samples are required for the electrochemical assessment of foodborne pathogens and contaminants.

As the preparation of food samples is ensured, one must take into account also its preservation prior its analysis, as the storage can last from several minutes up to days or even months. Several strategies may be applied to overcome physical, chemical, enzymatic, or microbial changes. The simplest treatment to overcome physical changes is temperature-controlled storage. To prevent oxidation of unsaturated lipids, dark containers, storage under nitrogen airflow, temperature-controlled environment, or the addition of antioxidants may be used. By heat and chemical treatment, drying, or freezing, both enzymatic inactivation and prevention of microbial contamination are ensured. Although lyophilization represents an election preservation method that extends the shelf-life of foodstuffs’ original properties, it is one of the most energy-consuming procedures [47].

## 5. Electrochemical Peptide-Based Sensors for Foodborne Pathogens Detection

Foodborne diseases are caused by different types of pathogens such as microbes, bacteria, and fungi, and they represent huge threats to human health. Thus, it is very important to ensure rapid and early-stage detection of these pathogens for preventing some severe diseases. In this regard, the use of sensors and biosensors for the identification and quantification of foodborne pathogens represents a current field of interest in research, this being mainly due to the high sensitivity, the fast quantification without the need of pretreatment, and the possibility of on-site testing with the help of simple, easy-to-use, and low-cost devices. An important class of peptides frequently used in the production of sensors is antimicrobial peptides (AMPs), small molecules (containing between six and 50 amino acid residues) discovered at the beginning of the 20th century which can be found in the immune systems of many organisms such as insects, amphibians, plants, microorganisms, and humans. AMPs are actually the first line in the body’s fight against microbes and pathogens such as bacteria, viruses, fungi, and cancerous cells.

AMPs began to be used as recognition elements in sensors due to their important features such as high stability, easy and stable synthesis strategies, and low costs. Several detection strategies for pathogen bacteria involving AMPs have been reported in the literature including electrochemical, optical, and piezoelectric ones, and most of these involve the use of nanomaterials and their integration on the transducers together with the appropriate peptide [29].

**Figure 4:**
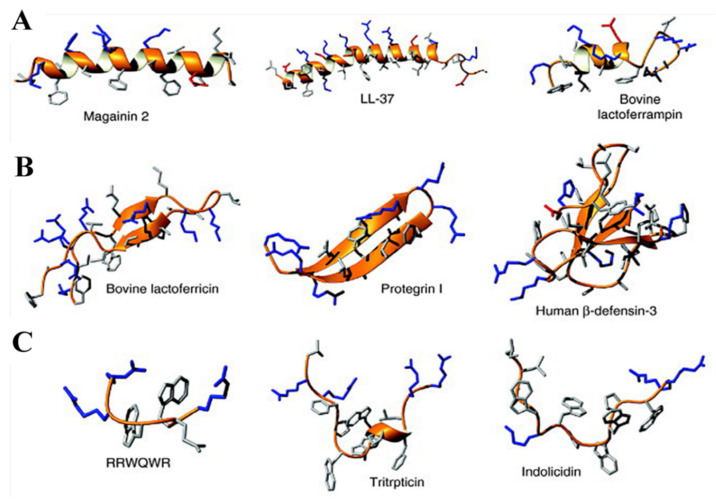
An overview of the major structural classes of AMPs. (**A**) α-Helical peptides, (**B**) β-sheet peptides, and (**C**) extended peptides. Copyright (2021) Elsevier [32].

The majority of AMPs present interactions with the cell membrane, this being followed by the disruption of cellular integrity. Due to this effect, the possibility of using AMPs in infectious therapy has been intensively studied, since the mechanism of action makes the emergence of resistances less likely compared to the antibiotics. Furthermore, AMPs might work as efficient alternative receptors for sensing applications. There are only some anionic AMPs, the vast majority having a cationic charge and a hydrophobic residue. There are three categories of cationic AMPs: linear α-helical AMPs; cysteine-rich AMPs; and extended AMPs enriched for specific AA, while the cytoplasmic membrane is the most frequent envisaged target [48]. Figure 4 presents some examples of AMP structures of all three types listed above. The structures presented here were solved by nuclear magnetic resonance (NMR) spectroscopy in the presence of detergent micelles, except for the β-sheet peptides, which were studied in aqueous solution. Positively charged side chains are colored in blue, negatively charged side chains are colored in red, and the remaining side chains in are colored in gray [32].

AMPs-based sensors involve the immobilization of the peptide on the substrates via electrostatic interactions; then, there is insertion into the cytoplasmic membrane when lipid bilayer disruption processes occur. This may result in constraints on the mobility of the peptides and of their capacity to transpose the cellular membrane. Due to limited experimental data on soluble and immobilized AMPs, it is difficult to determine the similarities and differences in the actions between immobilized AMPs and their soluble counterparts. For the elaboration of sensors, the high affinity of AMPs as recognition elements toward bacterial surface has attracted much more attention than their antimicrobial activity [29].

It was stated that the immobilized peptides are able to capture bacteria via electrostatic and hydrophobic interactions, but only few researchers have been devoted to studying the interactions between the immobilized AMPs and bacterial surface in biosensors. Even though the recognition mechanisms are unclear, to date, there are several thousand structures of natural AMPs elucidated and characterized in the databases, proving researchers’ interest in this subject.

As for the electrochemical sensors, methods such as amperometry, voltammetry, potentiometry, impedance spectroscopy, conductometry, and electrochemiluminescence were the most frequently used ones for foodborne pathogens detection [49].

### 5.1. Impedimetric Sensors

**Figure 5:**
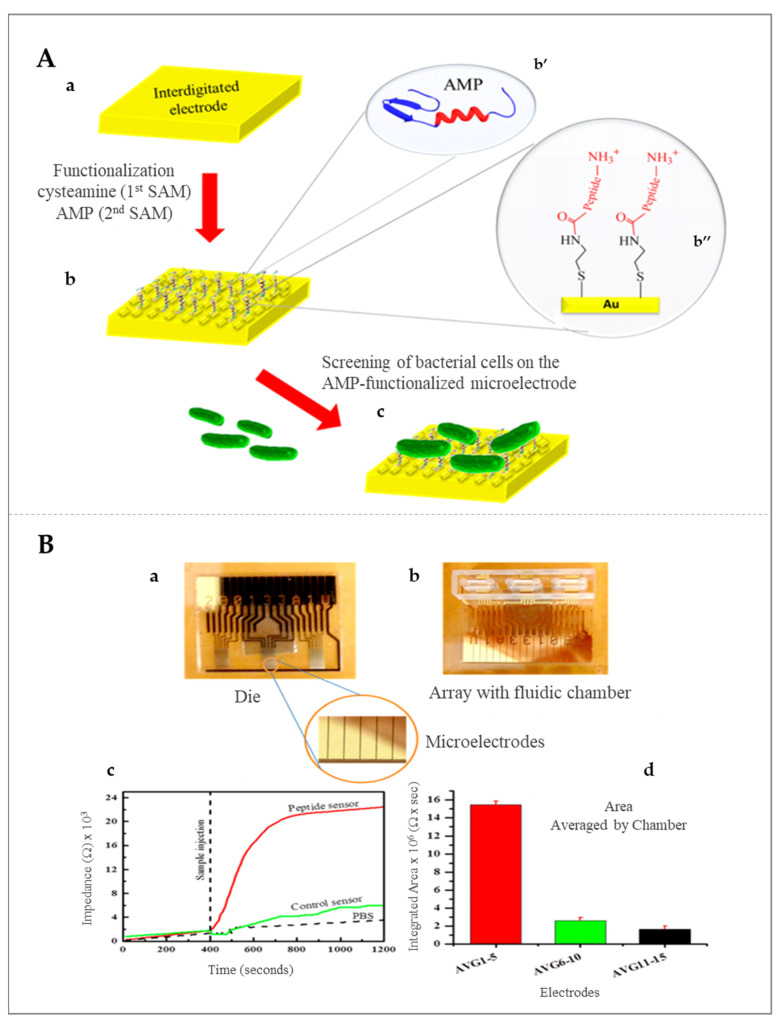
(**A**) Schematic representation of the AMP-based biosensor for *L. monocytogenes* detection at interdigitated microelectrode modified with antimicrobial peptides (AMP); (a) representation of the interdigitated electrode; (b) functionalization of the electrode with cysteamine AMP; (c) screening of bacterial cells. (**B**) Real-time measurements of binding of bacteria to the peptide sensor; (a) representation of the experimental assembly with the highlighting of the microelectrodes; (b) representation of array with fluidic chamber; (c,d) testing the peptide-based sensor. Copyright (2021) American Chemical Society [52].

Impedimetric sensors have more advantages as compared with other electrochemical sensors, being simple to operate, and which do not require special requirements such as the need for electroactive labels or enzymatic substrates. Different immobilization strategies were tested for peptides in order to improve the impedimetric signal. Thus, a magainin I-type peptide was immobilized on a microelectrode and applied for the impedimetric detection of *E. coli* Gram-negative bacteria, with good sensitivity and a limit of detection (LOD) of 10^3^ colony-forming unit (CFU) mL^−1^. Furthermore, it was demonstrated that the signal caused by pathogenic bacteria was much higher than in the case of nonpathogenic ones, which demonstrated that the proposed strategy may be suitable for the inter-bacterial strain differentiation as well as to discriminate between dead and live bacteria [50,51]. Gram-positive bacteria were also detected by electrochemical impedance, more precisely, *L. monocytogenes* was detected after the immobilization of Leucocin A, obtaining an LOD of 10^3^ CFU mL^−1^ in food samples. Schematic representation of the AMP-based biosensor for *L. monocytogenes* detection using an interdigitated microelectrode modified with AMP as well as the real-time measurements of binding of bacteria to the peptide sensor are presented in Figure 5 [52].

It was demonstrated that the selection of appropriate AMPs for the target bacteria plays an important role for the detection sensitivity. Thus, Jiang and coworkers have replaced magainin I into Colicin V for the detection of *E. coli*, and the obtained sensor allowed the impedimetric detection of the target bacteria as low as 10^2^ CFU mL^−1^ in contaminated water probes [53].

Further improvements in the detection could also be obtained by using nanomaterials together with peptides. Thus, carbon nanotubes (CNTs), graphene, carbon nanoparticles, and noble metals-based nanoparticles are some examples of nanomaterials, with unique physicochemical properties and notable mechanical strength, that are often applied to enhance the sensitivity and stability of the impedimetric peptide-based sensors [54]. The immobilization of CNTs on the surface of the electrode highly improves electron transduction of the electrode and facilitates the further immobilization of the peptide molecules for the target bacteria capture and detection. The use of gold nanoparticles (AuNPs) to replace CNTs in peptide-based sensors was also tested due to their simple synthesis, biocompatibility, and excellent conductivity [29].

### 5.2. Potentiometric Sensors

**Figure 6:**
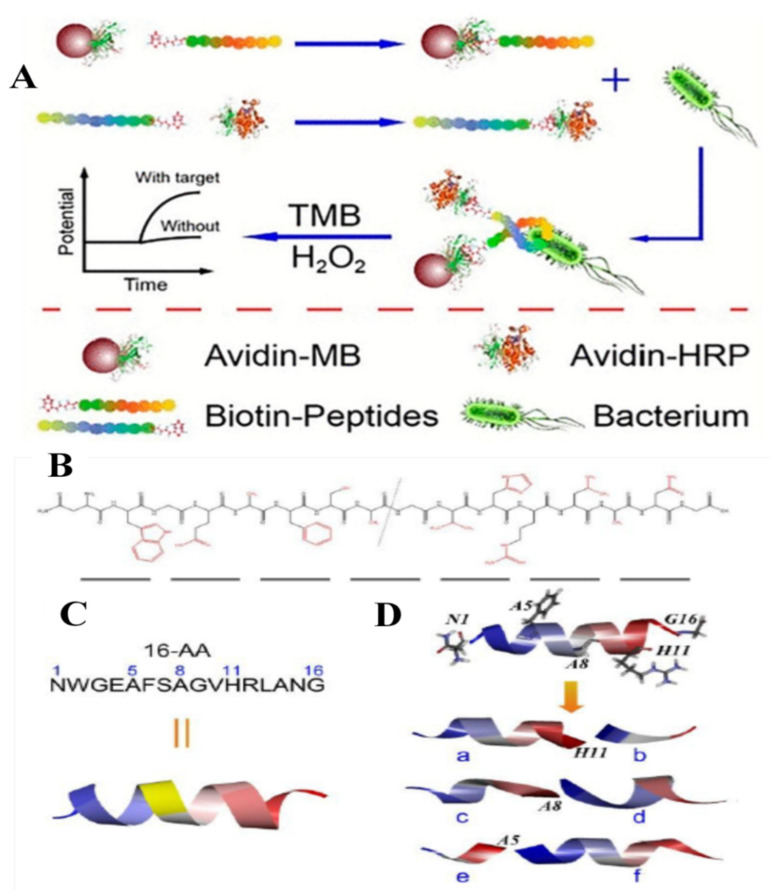
(**A**) Schematic representation of the potentiometric sandwich assay based on short AMP pairs for the detection of *L. monocytogenes*. (**B**) Structural formula of the original peptide. (**C**) Sequence and extended structure of the original peptide. (**D**) Calculated folded structures of the original peptide and synthesized split peptide pairs. Copyright (2021) American Chemical Society [55].

Potentiometric methods were also applied in the detection of foodborne pathogens via peptide-based sensors, these being the most sensitive detection strategies. For example, *L. monocytogenes* was detected by using an ion-selective polymeric membrane electrode and short antimicrobial peptide pairs as recognition molecules, namely two fragments derived from Leucocin A. The capture fragment was conjugated to magnetic beads to isolate target bacteria from the sample matrix. After being recognized and isolated by the modified magnetic beads as the capture fragment, bacteria were labeled by the detection fragment, which was conjugated with horseradish peroxidase (HRP) for catalyzing the substrate transformation (Figure 6A). The proposed potentiometric sensor can detect the target bacteria between 1.0 × 10^2^ and 1.0 × 10^6^ CFU mL^−1^ with an LOD of 10 CFU mL^−1^. A long peptide with 16 amino acids (WGEAFSAGVHRLAN) has been identified in this study to be specific for this bacterium. This peptide was split into two fragments to serve as the peptide for a sandwich assay. As can be observed in Figure 6B,C, the split peptides were designed according to the long peptide in order to maintain the excellent recognition ability. The calculated folded structures of the original peptide and the synthesized split peptide pairs are presented in Figure 6D (herein, chains of b, d, and f were used as the capture fragments, and chains of a, c, and e were used as the detection fragments) [55].

### 5.3. Voltammetric and Amperometric Sensors

Voltammetric techniques, such as cyclic voltammetry (CV), square wave voltammetry (SWV), or differential pulse voltammetry (DPV), have been intensively used during the last decades for the development of sensors and biosensors with several applications in food quality control and food safety domains. This is primarily due to the simplicity of these techniques as well as the high sensitivity and analytical performance that can be obtained rapidly and at low cost. Furthermore, the most important applications in this field of research are related with the detection of food resident contaminants (e.g., bacteria, viruses, and parasites) and the verification of the therapeutic ingredients of dietary supplements. The use of enzymes and the elucidation of the enzyme kinetics are other interesting domains in which the voltammetric techniques were found as effective analytical tools.

Amperometric sensors are based on the measurements of the electrical current developed from reductive and oxidative reactions and their correlation to the target analyte level in the samples. A fixed potential is applied to the transducer electrode to favor the redox reactions of the electroactive species from the bulk solution to the interface with the electrolyte. The most famous amperometric sensor has been the glucose sensor first described by Clark and Lyons in 1962, the mechanism of transduction being based on the oxidation of glucose mediated by the enzyme glucose oxidase into hydrogen peroxidase [56]. Working electrodes used in the development of amperometric biosensors are solid, relatively inert, highly conductive, and with low background currents. The most common electrodes used in amperometry are platinum, glassy carbon and gold for the anodic oxidation of hydrogen peroxide, whereas all types of carbon electrodes are applied for the anodic oxidation of nicotinamide adenine dinucleotide (NADH) and mediators. Another important requirement is related to the suitability of the electrodes for the functionalization via electrodeposition or chemical modification [57].

The development of novel electrode materials, which are made of composite (nano)materials that determine the improved characteristics and properties, allowed even higher sensitivity for the voltammetric-type electrochemical sensors. Thus, voltammetry has become an ideal methodology for testing the (bio)sensors and has the potential to serve as next-generation highly sensitive, robust, and selective analytical tools, enabling even multiplexed analysis, fast response, and cost-efficient studies in food monitoring and food safety domains [58].

The use of electrodes functionalized with nanomaterials has gained the attention of researchers because of their remarkable advantages such as increased peak current and decreased potential in voltammetry. Carbon-based nanostructured materials, such as carbon nanotubes, graphene, carbon quantum dots, fullerenes, etc. [59], metal nanoparticles such as AuNPs, silver nanoparticles (AgNPs), and platinum nanoparticles (PtNPs) [60], metal oxides such as oxides of copper, nickel, zinc, and iron [61], MIPs [39], or conductive polymers [62] have been applied in preparing electrochemical biosensors. The resulted remarkable electrochemical properties are usually due to the synergies between the basic electrode material and the applied modifiers.

An electrochemical DNA biosensor consisting of a selected aptamer immobilized onto a gold electrode surface functionalized with methylene blue was developed as the first peptide-based paper electrochemical sensor for the detection of botulinum neurotoxins. Botulinum neurotoxins produced by soil bacterium *Clostridium botulinum* are the cause of botulism and listed as biohazard agents, thus being necessary to quickly determine the presence of the bacterium in real samples. In this study, the presence of the neurotoxins was evaluated by monitoring the proteolytic activity through the measurement of the methylene blue electrochemical response. In fact, the analyte can cleave the portion of the peptide bound to methylene blue, leading to the decrease of the signal due to the removal of electrochemical probe from the working electrode surface. The biosensor based on the selected peptide and combined with a smartphone assisted potentiostat allowed the detection of the target analyte with a detection limit of 10 pM, obtaining excellent recoveries for spiked samples of orange juice [63].

**Figure 7:**
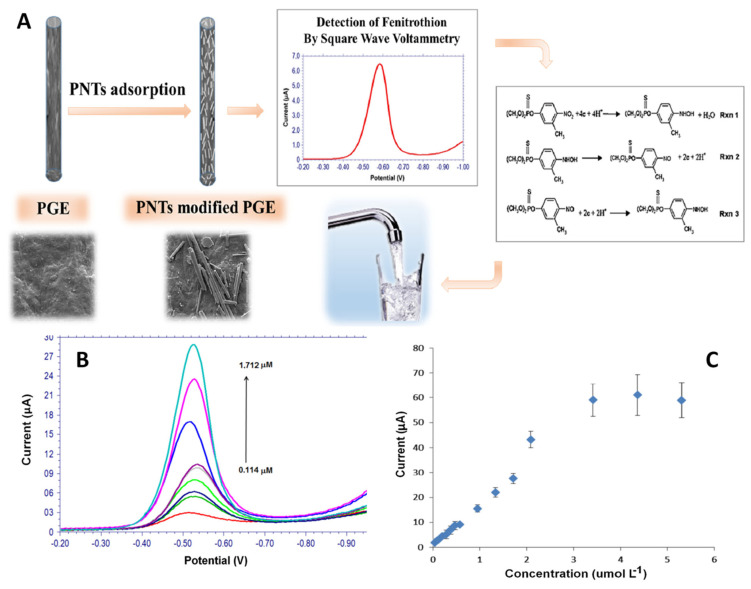
(**A**) Schematic representation of the elaboration and testing protocol of the electrochemical sensor for fenitrothion pesticide based on self-assembled peptide-nanotubes modified disposable pencil graphite electrode (PNT/PGE). (**B**) SWVs of fenitrothion pesticide at PNT/PGE for different concentrations from 0.114 to 1.712 μM. (**C**) Relationship between the peak currents and fenitrothion pesticide using PNT/PGE. Copyright (2021) Elsevier [64].

The sensitive voltammetric detection of fenitrothion was obtained using peptide-nanotubes (PNTs) as an electrode modifier for pencil graphite electrode (PGE). A linear correlation between the peak current and the concentration of the target analyte was obtained in the range from 0.114 μM to 1.712 μM with an estimated limit of detection of 0.0196 μM (S/N = 3) (Figure 7) [64].

### 5.4. Other Sensors

Optical biosensing methods such as colorimetry, fluorescence, and surface plasmon resonance (SPR) have been also widely applied as sensitive, easy to use, and fast methods for the detection of pathogens. The use of colorimetric detection is very attractive, because the result can be seen quickly and without any instrument, only with the naked eye [65]. The color change that usually occurs is due to a label, which may be an enzyme or metal nanoparticles such as AuNPs and AgNPs. The most widely used enzyme as a label is HRP, and the strategy refers to the initial obtaining of an enzyme–peptide conjugate, which is subsequently immobilized on the surface of target bacteria and catalyzes substrate transformation, causing a color change, and the intensity of this change is directly linked to the concentration of the target, and it can be applied for the quantification of the pathogens. For example, the HRP-peptide-based colorimetric detection of *E. coli* O157:H7 with an LOD of 13 CFU mL^−1^ in spiked apple juice and ground beef samples proved to be more sensitive than other methods [66]. The detection has been further simplified by using a colorimetric detection method based on peptide-functionalized magnetic nanoparticles and urease-catalyzed signal amplification. The bacteria were captured via the peptide-functionalized magnetic nanoparticles, the color change of the pH indicator being induced by the catalytic hydrolysis of urea into ammonium. Due to the high capture affinity, efficient amplification strategy, and simple manipulation, the proposed assay allowed the detection of *E. coli* O157:H7 in 30 min with a LOD of 12 CFU mL^−1^ [67].

Piezoelectric-based sensing methods are another promising and important tool for the real-time and label-free detection of foodborne pathogens. The working mechanism of piezoelectric methods is based on the transducing mass change induced by binding target bacteria into the change of frequency with piezoelectric crystals.

**Figure 8:**
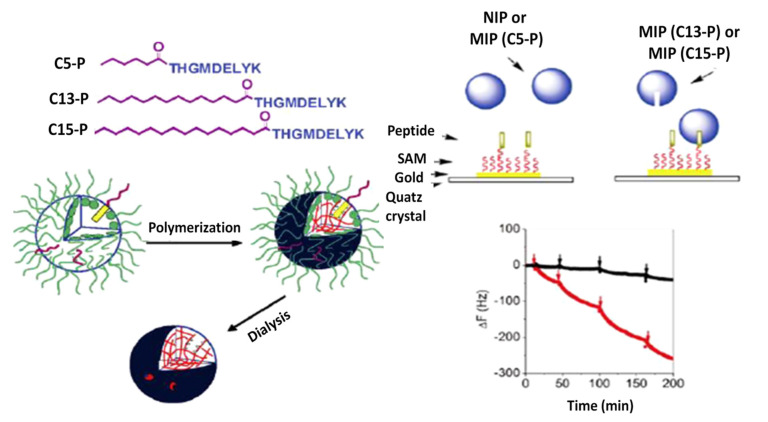
Schematic representation of the preparation protocol of the imprinted nanoparticles (**left**). Interaction between nanoparticles and immobilized peptide by QCM and the representative time courses of frequency change of the 27 MHz QCM (**right**). Copyright (2021) American Chemical Society [68].

The schematic representation of the preparation protocol of the imprinted nanoparticles (NPs) as well as the interaction between NPs and immobilized peptide C5-P, C13-P, and C15-P by QCM is presented in Figure 8. Frequency shift upon injections of NIP, MIP(C5-P), MIP(C13-P), and MIP(C15-P) to QCM sensor cells with GFP-9 immobilized on the surface proved the proper operation of the sensor [68].

In a typical AMPs-based piezoelectric method, AMPs are immobilized on the surface of the crystal to capture the target bacteria that enables the mass increasing. When the mass increases, the frequency of oscillation of the electrode will change, this change being correlated with the concentration of bacteria. An effective piezoelectric biosensor was developed where the analytical signal used was based on the dissociation of the peptides from the electrode surface, which determines the decrease of its mass, this decrease influencing the oscillation of the crystal. The immobilization of the peptide units was done via the adsorption on the surface of single-walled carbon nanotubes (SWCNTs), which were deposited on the surface of the crystal. In contact with the target bacteria, the peptide detaches from the outer surface of SWCNT, resulting in the frequency shift response of the piezoelectric sensor. The developed method could achieve fast and sensitive label-free detection of different bacterial strains [69].

The integration of microfluidics elements in detection systems is becoming an attractive alternative, as microbead-based microfluidic devices are widely used in bioassay development. Several applications were reported especially in the detection of *E. coli* using microchannels filled with AMPs-labeled beads designed to capture the bacteria strains, or microfluidic systems with AMPs modified with fluorophore as the label. Fluorescence microscopy or isotachophoresis were used to detect bacteria in water samples without any sample pretreatment. The peptide-based microfluidic systems developed so far for foodborne pathogens have low sensitivity and have complex instrumentation, which is mainly due to the required detection system. Thus, some simple and easy to be used biosensing methods such as electrochemical and optical (colorimetric) integrating microfluidic systems are required [48].

More examples of peptide-based sensors for foodborne pathogens detection are presented in Table 1.

To sum up, the electrochemical signal is generated by the presence of the analyte on the electrode surface or by a direct formation of electroactive species by the target molecule (analyte) or indirectly by coupling a biorecognition event with a redox probe or a mediated enzyme electrode. Several measurements modes are available: amperometry, potentiometry, conductometry, and impedimetry.

On the other hand, optical biosensors provide an optical signal (e.g., fluorescence color or chemiluminescence) that is generated directly by a bioreceptor and biomarker or through a recognition process. The most known recognition events provided by the formation of an antibody–antigen complex can be measured by the optical biosensor using an antibody labeled with a fluorescent probe. A change in the optical properties of the environment could also be considered a recognition event, which does not directly generate an optical signal. Label-free detection methods of biological elements are SPR and surface-enhanced Raman spectroscopy.

The simplest optical method is the colorimetric one, which can be observed by the naked eye. In order to obtain a quantitative answer, the optical sensors must integrate a photodetector such as photodiodes, photomultipliers, or a CDD camera capable of converting the optical signal into a measurable electrical signal.

The main properties of optical biosensors are that they are fast, sensitive, reliable, and easily adaptable to multiplex format. They do have some disadvantages: susceptibility to environmental interferences that may cause photobleaching of photoactive molecules, and usually, there is a need for expensive filters and/or fragile optics.

On the other hand, the electrochemical biosensors offer fast and highly selective response, good sensitivity, and low cost. They are capable of real-time measurements and non-destructive sensing. In the last decades, some biosensors were reported to be more robust for long-term use. In order to reduce the time and the cost of analysis, some approaches related to their reuse were proposed. However, future challenges need to be faced by the optical and electrochemical sensors such as miniaturization and standardization [77].

When comparing those types of sensors, there are some important parameters that should be considered such as limit of detection, sensitivity, and limit of quantification, linear range, reproducibility, and selectivity. The repeatability, directly related to accuracy, is another important parameter that helps scientists repeat the measurements in the same experimental conditions. To ensure good selectivity and sensitivity, a low limit of detection is required that often increases the time to prepare the sensor, implying much more effort and money. A good example is the synthesis of colorimetric and fluorescent probes for optical sensors able to detect analytes in real samples, implying complicated procedures. If the electrode surface needs to be modified for electrochemical sensors, the stability of the modifier is an important issue that must be overcome.

However, the electrochemical sensors gained much attention due to their advantages: simplicity, sensitivity, good repeatability and reproducibility, long-term stability, cost effectiveness, excellent electrical conductivity, biocompatibility, large surface area, non-toxic properties, low cost of carbon-based nanomaterials, redox efficiency, and high conductivity.

Optical sensors have high reproducibility and sensitivity, while their detection limits are in the range of nanomolar. One huge advantage when using optical sensors is the lack of electrical wires because the optical signals are transmitted through optical fibers [78].

However, due to their inherent limitations, scientists developed hybrid techniques such as electrochemiluminescence (ECL), which combines electrochemistry with chemilumininescence. The chemical species that are generated at the surface of the electrodes undergo electron transfer reactions to form excited states that emit light. It became a powerful technique combining the features of electrochemical and optical sensors.

## 6. Conclusions and Future Trends

The affinity of peptides for organic and inorganic compounds, as well as chemical stability, biocompatibility, low immunogenicity, low cost, and simple synthesis protocols, are only some of the advantages and premises for their successful integration in the development of sensing devices for applications in many important domains, including the biomedical one. This type of devices based on electrochemical methods have the capacity to detect a specific target in a short time span, with high specificity, accuracy, without using complicated procedures of sampling or toxic and expensive reagents. Generally, the electrodes are of single use, which is an important requirement to diminish safety risks when analyzing food samples.

The development of electrochemical sensing devices became a mandatory condition in the alimentary industry to ensure the safety and quality of food and to reduce any unpredictable events along the alimentary food chain. As usual, the specific and rapid detection of various bacteria targets is of major importance when designing this kind of sensing device. The exhaustive development of nanomaterials with catalytic properties such as metallic/carbon-based nanomaterials or conductive polymers paired with a highly specific biorecognition element had a major impact in assessing food safety in different stages of productions and on the final product.

Peptides can adopt different configuration structures and can specifically recognize a molecule. The interactions between the two compounds allow the identification of the contaminant and the implementation of decontamination measures without altering the final product and reducing additional costs. The current stage of peptide-based sensors development does not allow their exclusive use in the alimentary industry, but the perspectives are more than promising to become complementary testing tools. Meanwhile, the conventional culture plating, PCR, and ELISA procedures represent the golden standard, and their performances are difficult to be overcome. The fact that electrochemical sensors could be integrated in portable point-of-use (POU) devices that allow the rapid, specific detection of markers for foodborne bacteria represents an important feature that enables their use with a high degree of confidence. In addition, the low consumption of reagents and decontamination protocols, which are significantly easier than the standard methods, must be taken into account. In this case, the sampling and testing could be performed even to evaluate the shelf life of the products allowing a fast analysis response and eliminating the transportation procedures that are also prone to supplementary contamination.

Economic impact is another issue that could not be ignored in the case of food industry where the use of rapid detection devices could reduce the cross-contamination risk and indirectly the implications for the healthcare system.

## Figures and Tables

**Table 1:** Comparison of some peptide-based detection methods for foodborne pathogenic bacteria.

Method	Target	Peptide	Sequence	LOD (CFU/mL)	Sample Type	Ref.
Electrochemical	*E. coli* O157:H7	Magainin I	GIGKFLHSAGKFGKAFVGEIMKS	10^3^	PBS	[70]
	*E. coli* O157:H7	Magainin I	GIGKFLHSAGKFGKAFVGEIMKS	10^3^	PBS	[50]
	*L. monocytogenes*	Leucocin A	KYYGNGVHCTKSGCSVNWGEAFSAGVHRLANGGNGFW	10^3^	10% milk	[52]
	Gram-negative bacteria	ClavA	VFQFLGKIIHHVGNFVHGFSHVF	10^2^	PBS	[71]
	*L. monocytogenes*	Leucocin A	KYYGNGVHCTKSGCSVNWGEAFSAGVHRLANGGNGFW	10	seawater	[72]
Fluorescent	*E. coli* O157:H7	Cecropin P1	SWLSKTAKKLENSAKKRISEGIAIAIQGGPR	10^3^	PBS	[73]
Colorimetric	*E. coli* O157:H7	Magainin I	GIGKFLHSAGKFGKAFVGEIMKS	119,451	spiked apple juice; ground beef	[66]
	*E. coli* O157:H7	Magainin I	GIGKFLHSAGKFGKAFVGEIMKS	84,233	spiked apple juice and ground beef	[67]
	*E. coli* O157, O26, and O111	Cecropin P1	SWLSKTAKKLENSAKKRISEGIAIAIQGGPR	10^6^; 10^5^	spiked ground beef	[74]
SPR	*E. coli* O157:H7	Magainin I	GIGKFLHSAGKFGKAFVGEIMKS	5.0 × 10^2^	water, fruit and vegetable juice	[75]
QCM	*E. coli* O157:H7	Magainin I	GIGKFLHSAGKFGKAFVGEIMKS	400	spiked drinking water	[76]

## Data Availability

Not applicable.

## Abbreviations

AA	amino acids
AIDS	acquired immunodeficiency syndrome
ACE2	angiotensin-converting enzyme 2
AMPs	antimicrobial peptides
AgNPs	silver nanoparticles
AuNPs-	gold nanoparticles
CFU	colony-forming unit
CNTs-c	arbon nanotubes
COVID 19	coronavirus disease
CV	cyclic voltammetry
DPV	differential pulse voltammetry
DNA.	deoxyribonucleic acid
ECL	Electrochemiluminescence
EIS	electrochemical impedance spectroscopy
ELISA	enzyme-linked immunosorbent assay
Fmoc	Fluorenylmethyloxycarbonyl
HRP	horseradish peroxidase
IgG	immunoglobulin G
IgM	immunoglobulin M
IUPAC	International Union of Pure and Applied Chemistry
LOD	limit of detection
MIPs	molecularly imprinted polymers
NADH	nicotinamide adenine dinucleotide
NMR	nuclear magnetic resonance spectroscopy
PBS	phosphate buffer saline
PCR	polymerase chain reaction
PGE	pencil graphite electrode
PNTs	peptide nanotubes
POU	point-of-use
PtNPs	platinum nanoparticles
RNA	ribonucleic acid
SARS	severe acute respiratory syndrome
SPR	surface plasmon resonance
SWCNTs	single-walled carbon nanotubes
SWV	square wave voltammetry
t-Boc	tert-butyloxycarbonyl
WHO	World Health Organization
